# Physiological adaptations to resistance exercise as a function of age

**DOI:** 10.1172/jci.insight.95581

**Published:** 2017-09-07

**Authors:** Bethan E. Phillips, John P. Williams, Paul L. Greenhaff, Kenneth Smith, Philip J. Atherton

**Affiliations:** 1 School of Medicine and; 2 School of Life Sciences, Medical Research Council Arthritis Research UK Centre for Musculoskeletal Ageing Research, University of Nottingham, Nottingham and Derby, United Kingdom.

**Keywords:** Metabolism, Muscle Biology, Amino acid metabolism, Skeletal muscle

## Abstract

**BACKGROUND.** The impact of resistance exercise training (RE-T) across the life span is poorly defined.

**METHODS.** To resolve this, we recruited three distinct age cohorts of young (18–28 years; **n** = 11), middle-aged (45–55 years; **n** = 20), and older (nonsarcopenic; 65–75 years; **n** = 17) individuals to a cross-sectional intervention study. All subjects participated in 20 weeks of fully supervised whole-body progressive RE-T, undergoing assessment of body composition, muscle and vascular function, and metabolic health biomarkers before and after RE-T. Individuals also received stable isotope tracer infusions to ascertain muscle protein synthesis (MPS).

**RESULTS.** There was an age-related increase in adiposity, but only young and middle-age groups demonstrated reductions following RE-T. Increases in blood pressure with age were attenuated by RE-T in middle-aged, but not older, individuals, while age-related increases in leg vascular conductance were unaffected by RE-T. The index of insulin sensitivity was reduced by RE-T in older age. Despite being matched at baseline, only younger individuals increased muscle mass in response to RE-T, and there existed a negative correlation between age and muscle growth; in contrast, increases in mechanical quality were preserved across ages. Acute increases in MPS (upon feeding plus acute RE-T) were enhanced only in younger individuals, perhaps explaining greater hypertrophy.

**CONCLUSION.** Our data indicate that RE-T offsets some, but not all, negative characteristics of ageing — some of which are apparent in midlife.

**FUNDING.** Biotechnology and Biological Sciences Research Council (BB/C516779/1).

## Abstract 2

Although resistance exercise training–induced hypertrophy is blunted with advancing age, this regimen offsets some negative characteristics of aging that are apparent in midlife.

## Introduction

The mechanisms driving skeletal muscle atrophy and dysfunction with ageing have been the subject of intense study. A number of factors have been suggested, including physical inactivity (1) and more intrinsic factors of ageing, such as epigenetic changes (2), oxidative stress (3), inflammation (4), DNA damage, and mitochondrial dysfunction (5, 6). However, one known major driver of age-related muscle atrophy is so-called anabolic resistance (7, 8). This describes the phenomena whereby the main signals that maintain muscle mass and metabolic homeostasis, irrespective of age, namely nutrition (9, 10) and physical activity (8, 11–13), become dysregulated. Specifically, when comparing the acute anabolic responses to feeding and exercise in younger versus older individuals, responses are lesser in the old. On a day-to-day and chronic basis, “anabolic resistance” has therefore been suggested as a potential driver of sarcopenia.

Resistance exercise training (RE-T) arguably remains the most potent nonpharmacological anabolic stimulus for skeletal muscle and has been shown to enhance muscle mass and function in healthy younger (14), aged (15), and cachectic populations (16). Nonetheless, when looking at comparative gains in muscle mass following an allied program of supervised RE-T, muscle hypertrophic responses are typically blunted in older versus younger individuals. While the mechanisms for this remain incompletely defined, this is at least in part as a result of impaired ribosomal biogenesis and long-term muscle protein synthesis (MPS) (8, 17, 18). Indeed, a meta-analysis on this topic showed that the gains in lean mass at an older age are modest (~1 kg) compared with what is observed at younger age (19). The age at which this diminution in the capacity for muscle hypertrophy manifests remains undefined. Moreover, whether such anabolic resistance is only a feature of sarcopenia or is also present in healthy older people with normal muscle mass is not known.

Ageing is also associated with a host of other deleterious changes in body composition and metabolic health biomarkers. For instance, along with declines in lean mass, ageing is associated with increased adiposity (20), declines in bone mineral density (BMD) (21), reductions in insulin sensitivity (22), and reductions in vascular function (23) (e.g., endothelial function/arterial stiffness and increased blood pressure [BP]). Crucially, many of these facets have been shown to be modifiable by RE-T. For example, RE-T has been shown to reduce adiposity (24), lower BP (25), improve insulin sensitivity (26), and enhance BMD (27). Nonetheless, many of the studies showing these benefits have been conducted either in healthy groups of individuals (in specific age groups) or in relation to ameliorating the negative effects of metabolic diseases (e.g., type 2 diabetes) or obesity. Indeed, to our knowledge, there has not been a prospective longitudinal study of RE-T across age, i.e., contrasting responses of younger, middle-aged, and older healthy individuals.

**Figure 1:**
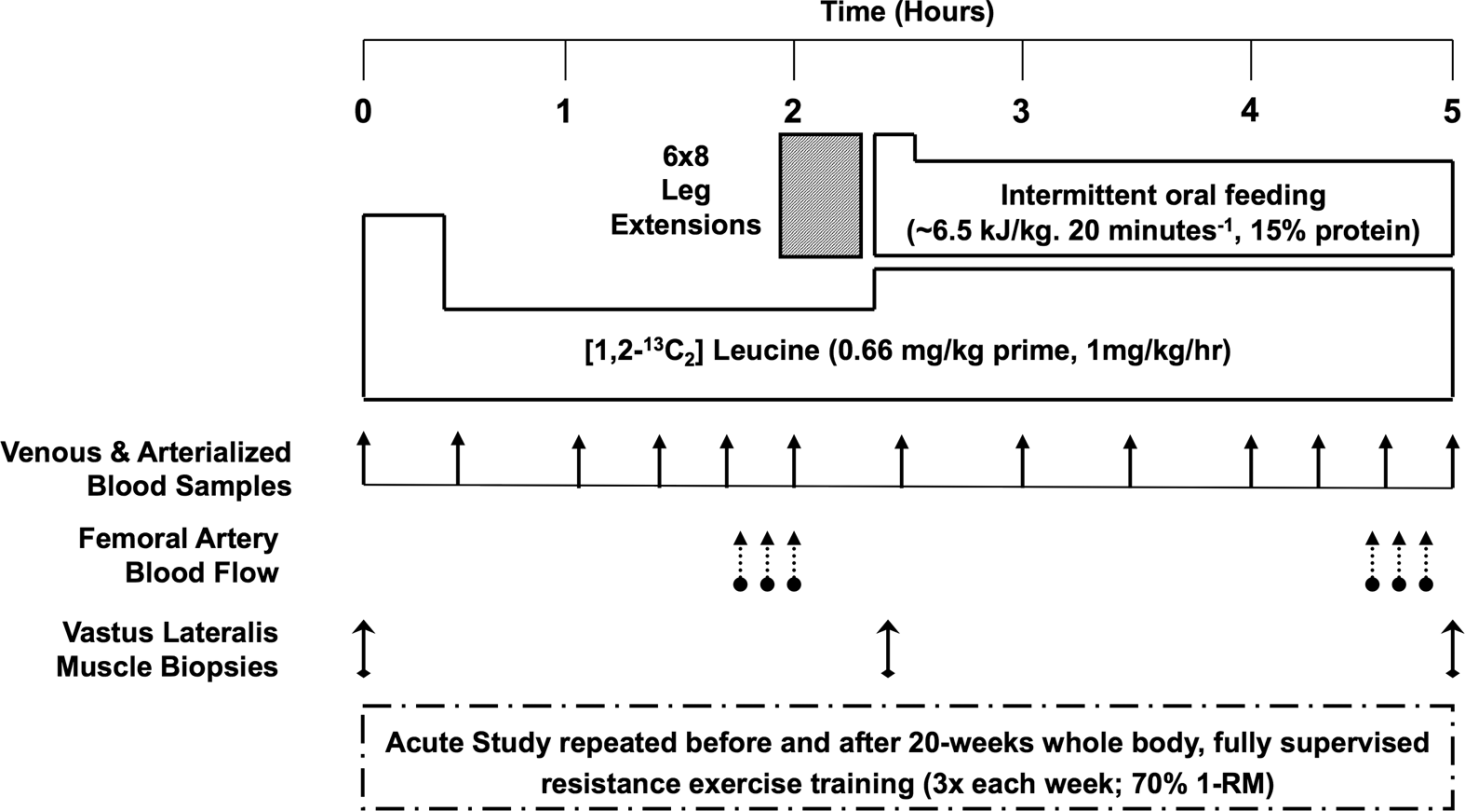
Acute study schematic. 1-RM, 1-repetition maximum.

To address this gap, we recruited 48 men and women who formed three groups, representing young, middle-aged, and older ages. All individuals were physiologically and metabolically phenotyped during an acute study day at baseline and following 5 months of fully supervised and progressive whole-body (WB) RE-T (Figure 1). At screening, we ensured each age group was well-matched in terms of lean mass and free of overt disease to preclude any influence of sarcopenia or conditions that may interfere with responses to RE-T as a function of age (e.g., there exists a negative association between insulin resistance and muscle mass, ref. 22).

## Results

### Lean mass and hypertrophic responses to RE-T

**Figure 2:**
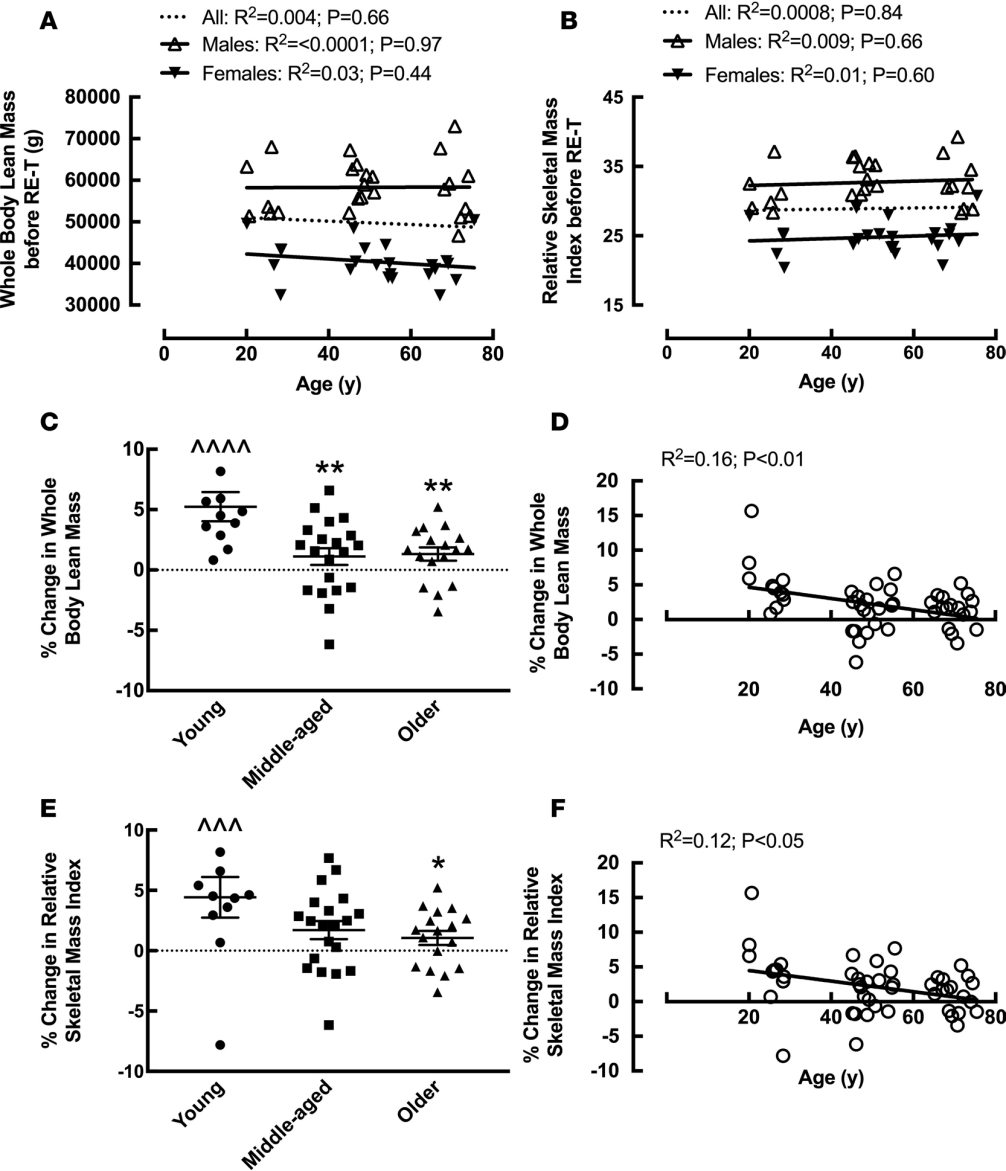
The effects of age and 20-week whole-body fully supervised resistance exercise training on lean mass. Whole body lean mass (**A**) and relative skeletal mass index (**B**) with advancing age before resistance exercise training (RE-T). Percentage change in whole body lean mass in young, middle-aged, and older individuals (**C**) and with advancing age (**D**) in response to RE-T. Percentage change in relative skeletal mass index in young, middle-aged, and older individuals (**E**) and with advancing age (**F**) in response to RE-T. Data for **C** and **E** are shown as mean ± SEM with individual data points. *n* = 11 young, 20 middle-aged, and 17 older individuals. Within and between group analysis via 2-way ANOVA with Tukey’s post-hoc analysis. Relationship analysis via Pearson’s correlation. ^^^*P* < 0.01; ^^^^*P* < 0.001 before RE-T vs. after RE-T; *P < 0.05; **P < 0.01 vs. young.

Despite no age-related differences in WB lean mass, leg lean mass, or relative skeletal mass index (RSMI: lean mass [kg]/height [m]) among the age groups (Table 1), or with age upon correlation analysis (Figure 2, A and B) before RE-T, hypertrophic responses to RE-T were blunted with advancing age. When comparing among the age groups, only young individuals demonstrated significant WB hypertrophy in response to RE-T (49,921 ± 3,065 g vs. 52,591 ± 3,347 g, *P* < 0.0001), with significantly greater gains in WB lean mass (5.24% ± 1.21%) than both middle-aged (1.12% ± 0.70%) and older (1.33% ± 0.55%) individuals (both *P* < 0.01) (Figure 2C). Assessing the relationship between advancing age and WB hypertrophic responses, there was a significant negative correlation between advancing age and ensuing WB hypertrophic responses (R^2^ = 0.16, *P* < 0.01) (Figure 2D). Similarly, when assessing RE-T–induced changes in RSMI, there was a significant negative correlation between advancing age and RE-T–induced changes in RSMI (R^2^ = 0.12, *P* < 0.05) (Figure 2F), with only young individuals demonstrating a significant increase in RSMI in response to RE-T (28.09 ± 1.43 vs. 29.39 ± 1.66, *P* < 0.01). The increase in RSMI in young individuals was significantly greater than that achieved by older individuals (4.43% ± 1.69% vs. 1.06% ± 0.57%, *P* < 0.05) (Figure 2E). When assessing leg hypertrophic responses, again only the young individuals demonstrated a hypertrophic response to RE-T (8,780 ± 533 g vs. 9,154 ± 525 g, *P* < 0.05) (Table 1). However, due to heightened heterogeneity in leg hypertrophic responses (95% CI, leg: –4.70%–8.94%; WB: –2.68%–6.93%), there was no correlation between advancing age and leg hypertrophic responses to RE-T. Considering sex differences, there was no significant relationship between advancing age and WB lean mass (Figure 2A) or RSMI (Figure 2B) for either men or women before RE-T.

### Physical function and alterations with RE-T

**Figure 3:**
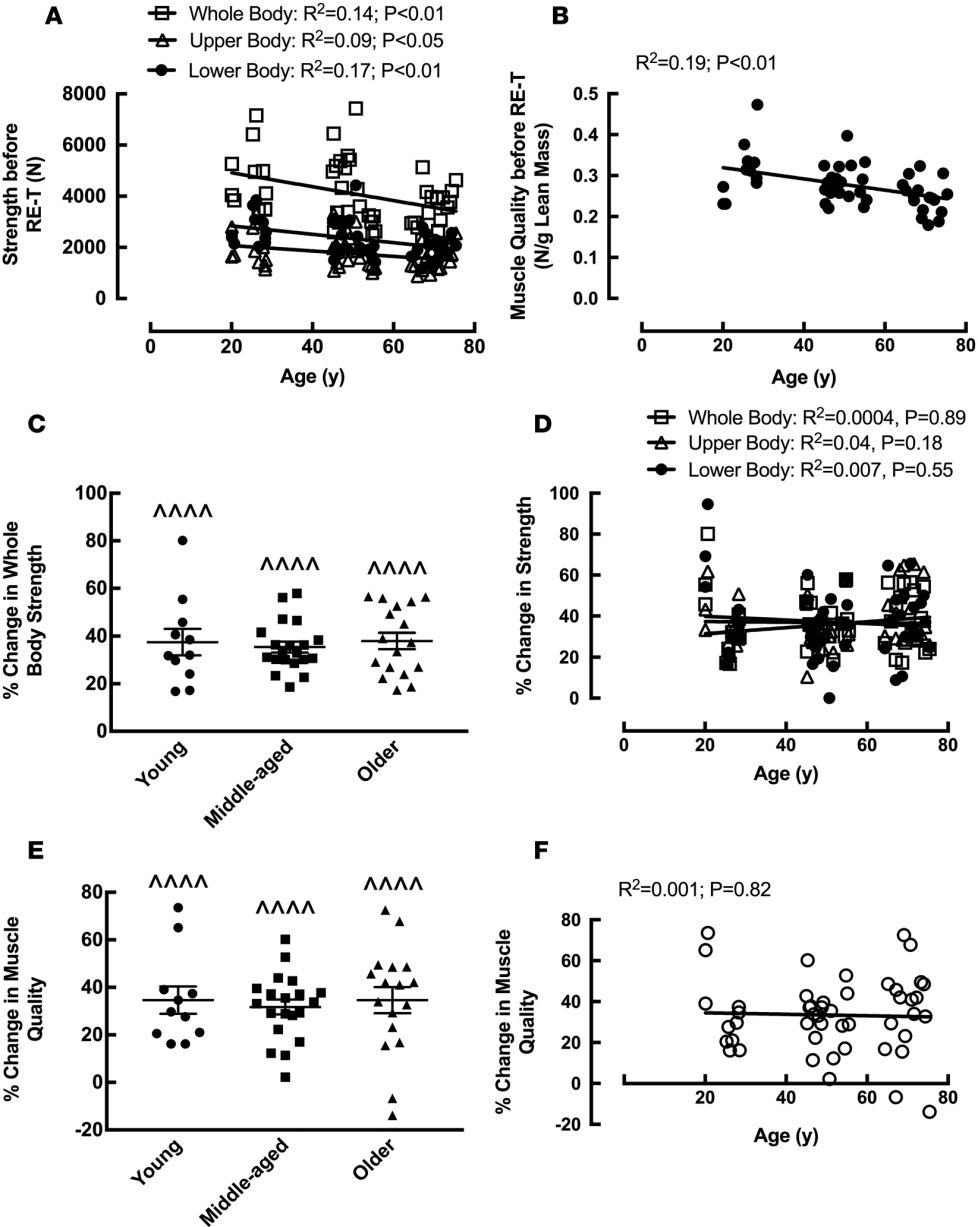
The effects of age and 20-week whole-body fully supervised resistance exercise training on physical function and muscle quality. Whole body and upper and lower body strength (**A**) and muscle quality (leg strength in newtons [N] per unit lean leg mass [g]) (**B**) with advancing age before resistance exercise training (RE-T). Percentage change in strength (whole body) in young, middle-aged, and older individuals (**C**) and with advancing age (whole body, upper body, and lower body) (**D**) in response to RE-T (**B**). Percentage change in (leg) muscle quality in young, middle-aged, and older individuals (**E**) and with advancing age (**F**) in response to RE-T. Data for **C** and **E** are shown as mean ± SEM with individual data points. *n* = 11 young, 20 middle-aged, and 17 older individuals. Within and between group analysis via 2-way ANOVA with Tukey’s post-hoc analysis. Relationship analysis via Pearson’s correlation. ^^^^*P* < 0.001 before RE-T vs. after RE-T.

Similar to the data for WB lean mass, there were no significant age-related differences in WB strength before RE-T (despite a trend [*P* = 0.08] for the young to be significantly stronger than the old) (Table 1). This was also true for upper body strength (Table 1). The young group did, however, have significantly greater lower body strength than the older age group before RE-T (2,706 ± 184 vs. 1,991 ± 107 N, *P* < 0.05) (Table 1). Mirroring the results for lower body muscle strength, lower limb muscle quality was significantly lower in the older age group compared with the younger group before RE-T (0.31 ± 0.02 N/g vs. 0.25 ± 0.01 N/g, *P* < 0.01) (Table 1). Upon correlation analysis, a significant negative relationship was observed between advancing age and all three facets of strength (WB [R^2^ = 0.14, *P* < 0.01], upper body [R^2^ = 0.09, *P* < 0.05, and lower body [R^2^ = 0.17, *P* < 0.01]) before RE-T (Figure 3A). This was also apparent for muscle quality (R^2^ = 0.19, *P* < 0.01) (Figure 3B). There were, however, no significant differences in WB (Figure 3C), upper body, or lower body strength gains made among the age groups (all *P* < 0.0001), with the young group being significantly stronger than the older group (whole [6,293 ± 405 N vs. 4,832 ± 294 N, *P* < 0.05] and lower [3,744 ± 185 N vs. 2,764 ± 179 N, *P* < 0.01] body) after RE-T (Table 1). There was no significant correlation between advancing age and any facet of strength improvement (Figure 3D). Changes in muscle quality were similar to those of strength, with no significant differences in muscle quality gains among the age groups (all *P* < 0.0001) (Figure 3E) and no significant relationship between advancing age and changes in muscle quality (Figure 3F).

### Body composition and alterations with RE-T

#### WB adiposity.

**Figure 4:**
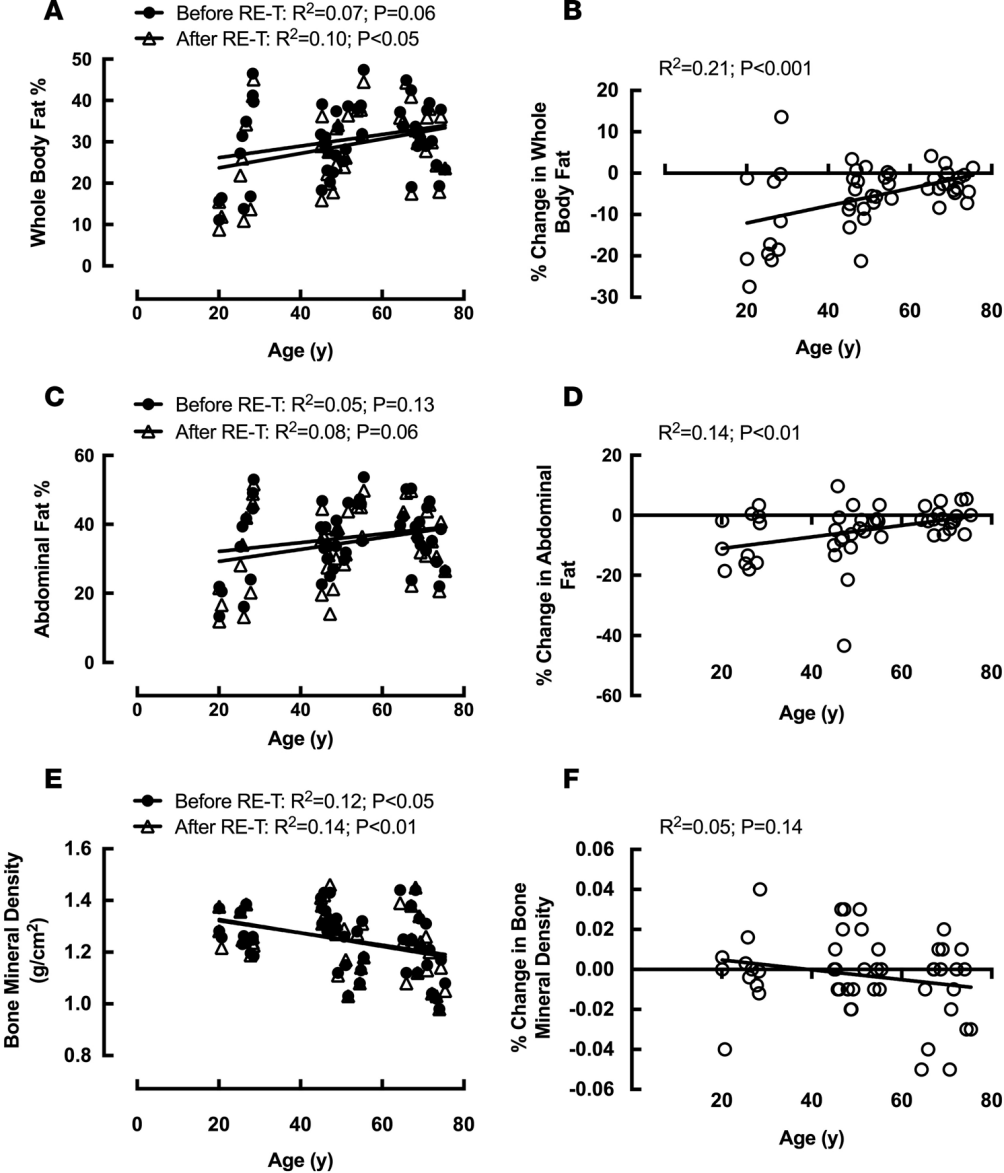
The effects of age and 20-week whole-body fully supervised resistance exercise training on body composition parameters. Whole body (**A**) and abdominal (**C**) fat mass and bone mineral density (**E**) with advancing age before and after resistance exercise training (RE-T). Percentage change in whole body (**B**) and abdominal (**D**) fat mass and bone mineral density (**F**) with advancing age in response to RE-T. *n* = 48 individuals. Relationship analysis via Pearson’s correlation.

There were no significant differences in WB fat mass among the age groups before RE-T (Table 1), despite a trend for increasing WB adiposity with advancing age (R^2^ = 0.07, *P* = 0.06) (Figure 4A). Reductions in WB adiposity with RE-T were apparent for the young (–11.4% ± 3.7%, *P* < 0.01) and middle-aged (–5.0% ± 1.3%, *P* < 0.01) groups only (Table 1), with significantly greater losses in the young group when compared with the older age group (–11.4% ± 3.7% vs. –2.37% ± 0.79%, *P* < 0.01). Advancing age was associated with reduced WB RE-T–induced adipose losses (R^2^ = 0.21, *P* < 0.001) (Figure 4B). However, despite this age-related attenuation of body fat losses with RE-T, there still existed no significant differences in WB adiposity among the age groups after RE-T (Table 1). There was, however, a significant relationship between advancing age and WB adiposity after RE-T (R^2^ = 0.10, *P* < 0.05) (Figure 4A).

#### Abdominal adiposity.

Similar to the data for WB adiposity, there were no significant differences in abdominal (Ab) fat mass among the age groups before RE-T (Table 1), with no significant relationship between Ab adiposity and advancing age (Figure 4C). Only the young (–8.6% ± 2.5%, *P* < 0.05) and middle-aged (–6.7% ± 2.4%, *P* < 0.001) groups demonstrated significant reductions in Ab fat mass with RE-T (Table 1), with advancing age associated with reduced RE-T–induced Ab adipose losses (R^2^ = 0.14, *P* < 0.01) (Figure 4D). However, as with WB adiposity, despite an age-related attenuation of body fat losses with RE-T, there still existed no significant differences in Ab adiposity among the age groups after RE-T (Table 1). There was, however, a trend toward a significant relationship between advancing age and Ab adiposity after RE-T (R^2^ = 0.80, *P* = 0.06) (Figure 4C).

#### BMD.

Despite there being no significant differences in BMD among the age groups before or after RE-T (Table 1), there was a significant negative correlation between advancing age and BMD both before (R^2^ = 0.12, *P* < 0.05) and after RE-T (R^2^ = 0.14, *P* < 0.01) (Figure 4E). None of the groups showed RE-T–induced changes in BMD, with no difference in change among the age groups (Table 1) and no relationship between age and RE-T–induced changes in BMD (Figure 4F).

### WB health indices and alterations with RE-T

#### Cardiovascular parameters.

**Figure 5:**
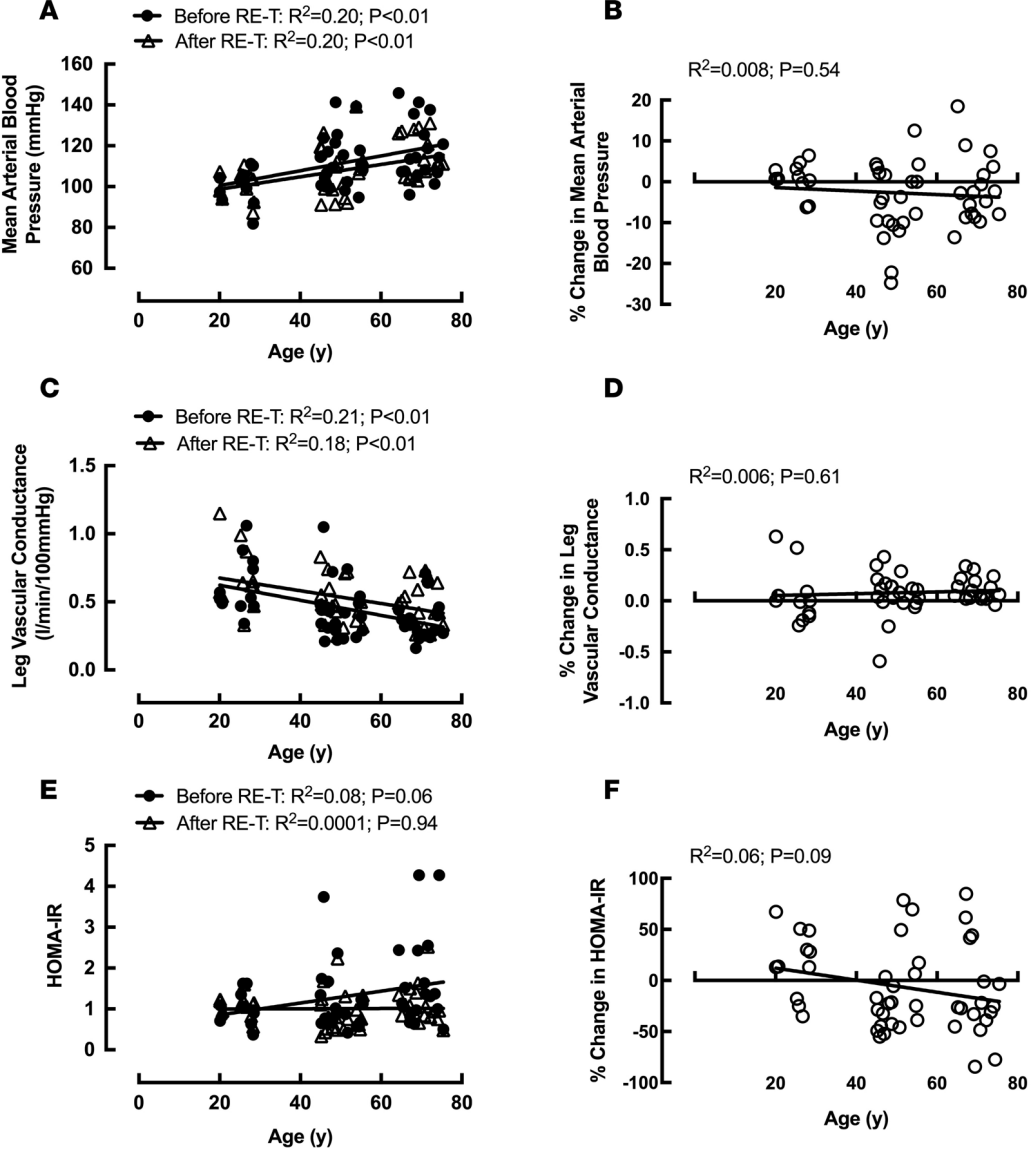
The effects of age and 20-week whole-body fully supervised resistance exercise training on parameters of whole-body health. Mean arterial blood pressure (**A**), leg vascular conductance (**C**), and homeostasis model assessment of insulin resistance (HOMA-IR) (**E**) with advancing age before and after resistance exercise training (RE-T). Percentage change in mean arterial blood pressure (**B**), leg vascular conductance (**D**), and HOMA-IR (**F**) with advancing age in response to RE-T. *n* = 48 individuals. Relationship analysis via Pearson’s correlation.

Systolic BP (SBP), diastolic BP (DBP), and mean arterial (blood) pressure (MAP) were significantly greater in older individuals compared with young individuals before RE-T (SBP: 139 ± 4 mmHg vs. 118 ± 3 mmHg; DBP: 79 ± 3 mmHg vs. 68 ± 2 mmHg; MAP: 118 ± 4 mmHg vs. 100 ± 3 mmHg, all *P* < 0.01) (Table 1; MAP only). All three BP parameters were also significantly greater in the middle-aged group (SBP: 132 ± 4 mmHg, *P* < 0.05; DBP: 79 ± 2 mmHg, *P* < 0.01; MAP: 113 ± 3 mmHg, *P* < 0.05) compared with the young group (Table 1; MAP only). For all three BP parameters, there was a significant relationship between advancing age and BP before RE-T (SBP: R^2^ = 0.17, DBP: R^2^ = 0.15, MAP: R^2^ = 0.20, all *P* < 0.01) (Figure 5A; MAP only). No significant changes in SBP were observed in any age group following RE-T, with significant reductions in DBP (79 ± 2 mmHg vs. 73 ± 2 mmHg, *P* < 0.05) and MAP (113 ± 3 mmHg vs. 107 ± 3 mmHg, *P* < 0.05) in the middle-aged group only (Table 1; MAP only). There were no significant relationships between advancing age and RE-T–induced changes in any of the BP parameters (Figure 5B; MAP only). After RE-T, SBP (135 ± 3 mmHg vs. 118 ± 3 mmHg, *P* < 0.05) and MAP (115 ± 2 mmHg vs. 101 ± 2 mmHg, *P* < 0.01) remained greater in the older age group compared with the young age group, with no significant differences in DBP among the age groups (Table 1; MAP only). The correlation between advancing age and BP parameters remained after RE-T (SBP: R^2^ = 0.18, DBP: R^2^ = 0.11, MAP: R^2^ = 0.20, all *P* < 0.01) (Figure 5A; MAP only).

Leg vascular conductance (LVC) was significantly greater in young individuals compared with middle-aged (0.62 ± 0.07 l/min·100 mmHg vs. 0.43 ± 0.05 l/min·100 mmHg, *P* < 0.05) and older (0.36 ± 0.03 l/min·100 mmHg, *P* < 0.001) individuals before RE-T (Table 1), with a significant negative relationship between age and LVC (R^2^ = 0.20, *P* < 0.01; Figure 5C). None of the age groups demonstrated significant improvements in LVC in response to RE-T (Table 1), with the negative relationship between age and LVC remaining after RE-T (R^2^ = 0.18, *P* < 0.01) (Figure 5C). There was no relationship between advancing age and RE-T–induced changes in LVC (Figure 5D). After RE-T, the young age group (0.68 ± 0.07 l/min·100 mmHg) still displayed significantly greater LVC than both the middle-aged (0.50 ± 0.04 l·min^–1^·100mmHg^–1^) and older (0.47 ± 0.04 l/min·100 mmHg) age groups (both *P* < 0.05) (Table 1).

#### Fasting cholesterol and triglycerides.

There were no significant differences in total cholesterol, LDL cholesterol, or HDL cholesterol among the age groups either before or after RE-T. No significant RE-T–induced changes were seen in any of these parameters, with no significant differences in changes among the age groups (Table 1). There were no significant relationships between age and total cholesterol or HDL cholesterol either before or after RE-T, although a significant relationship between advancing age and LDL was observed before RE-T only (R^2^ = 0.08, *P* < 0.05). Levels of plasma triglycerides were not significantly different among the age groups before RE-T (Table 1). Despite no significant changes in triglycerides in any age group, and no significant differences in changes among the age groups, a significant relationship between age and triglycerides was apparent after RE-T only (R^2^ = 0.08, *P* < 0.05).

#### Fasting insulin and glucose.

There were no significant differences in fasting insulin values among the age groups either before or after RE-T (Table 1). Similarly, at neither time point did fasting insulin correlate with age. None of the age groups demonstrated a significant reduction in fasting insulin after RE-T (Table 1), with no relationship between changes in fasting insulin with RE-T and age.

Before RE-T, fasting glucose was significantly higher in the older age group compared with the young age group (5.85 ± 0.11 mmol/l vs. 5.17 ± 0.16 mmol/l, *P* < 0.05) (Table 1), despite no significant correlation between fasting glucose and advancing age. Only the older age group demonstrated a significant reduction in fasting glucose with RE-T (5.85 ± 0.11 mmol/l vs. 5.33 ± 0.21 mmol/l, *P* < 0.01), abolishing the significant difference in fasting glucose between young and older subjects that was seen before RE-T (Table 1). There was no relationship between age and RE-T–induced changes in fasting glucose.

Employing the homeostasis model assessment (HOMA) as an index of insulin resistance (IR), older individuals had significantly greater HOMA-IR (1.70 ± 0.28) than young (1.01 ± 0.12) and middle-aged (1.13 ± 0.17, both *P* < 0.05) individuals before RE-T (Table 1), with a trend for a correlation between advancing age and HOMA-IR (R^2^ = 0.08, *P* = 0.06) (Figure 5E). Only older individuals showed a significant reduction in HOMA-IR after RE-T (1.70 ± 0.28 vs. 1.11 ± 0.12, *P* < 0.05), such that the trend for a relationship between advancing age and HOMA-IR was not apparent after RE-T (R^2^ = 0.0001; *P* = 0.94) (Figure 5E). There were no significant differences in HOMA-IR among the age groups after RE-T (Table 1). There was a trend for a relationship between advancing age and reductions in HOMA-IR (R^2^ = 0.06; *P* = 0.09) (Figure 5F), with a highly significant correlation between HOMA-IR before RE-T and RE-T–induced reductions in HOMA-IR (R^2^ = 0.34; *P* < 0.0001), irrespective of age.

### MPS and RE-T

**Figure 6:**
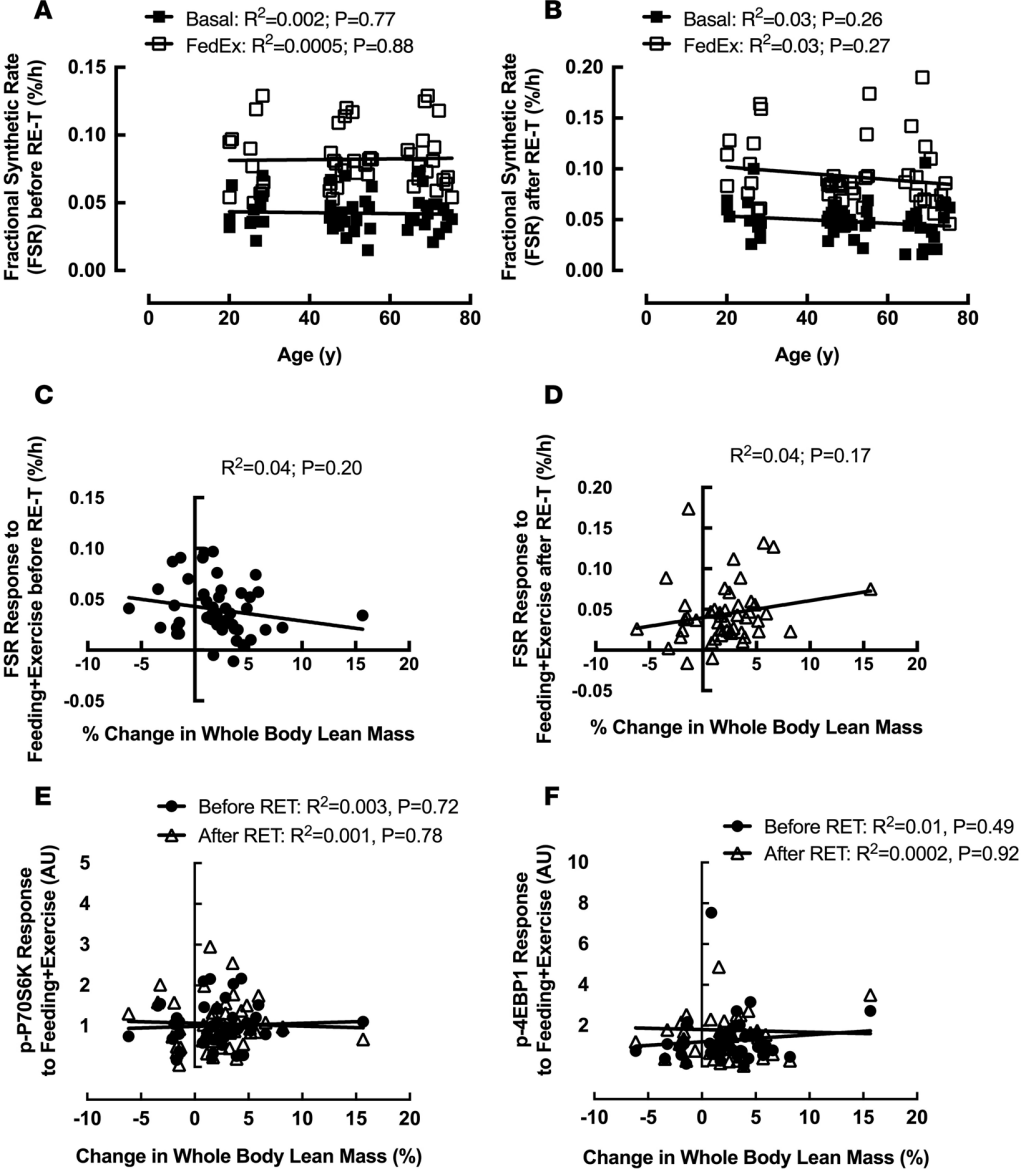
Relationships between baseline characteristics and resistance exercise training–induced hypertrophic responses after 20-week whole-body fully supervised resistance exercise training. Basal (fasted and rested) fractional synthetic rate (FSR) and FSR in response to feeding plus acute exercise (FedEx; 6 × 8 leg extensions) with advancing age before (**A**) and after (**B**) resistance exercise training (RE-T). The relationship between FSR response to FedEx and RE-T–induced muscle hypertrophy before (**C**) and after RE-T (**D**). The relationship between anabolic signaling responses (P70S6K [**E**]; 4EBP1 [**F**]) to FedEx and RE-T–induced muscle hypertrophy before and after RE-T. *n* = 48 individuals. Relationship analysis via Pearson’s correlation.

Basal fractional synthetic rate (FSR) was not significantly different among the age groups either before or after RE-T (Table 2), with no correlation between age and basal FSR at either time (Figure 6, A and B). Basal FSR was not altered in any of the age groups in response to RE-T (Table 2), with no relationship between advancing age and changes in basal FSR (R^2^ = 0.03; *P* = 0.24).

Similar to the results observed for basal FSR, FSR in response to acute exercise plus feeding (FedEx) was not significantly different among the age groups before or after RE-T (Table 2), with no correlation between age and FedEx FSR at either time (Figure 6, A and B). Only young individuals showed a significantly higher FedEx FSR after RE-T compared with that before (0.08 ± 0.01 % per h vs. 0.11 ± 0.01 % per h, *P* < 0.05) (Table 2), with no significant relationship between advancing age and RE-T–induced changes in FedEx FSR (R^2^ = 0.04; *P* = 0.15).

The increase in FSR in response to FedEx (FedEx FSR minus basal FSR) was not significantly different among the age groups before or after RE-T (Table 2), with no correlation between FSR response and age at either time (before RE-T: R^2^ = 0.002, *P* = 0.78; after RE-T: R^2^ = 0.004, *P* = 0.68). None of the groups showed changes in FSR response after RE-T compared with before (Table 2), and there was no relationship between age and RE-T–induced changes in FSR response to FedEx (R^2^ = 0.04; *P* = 0.16).

### Relationships between baseline status and ensuing hypertrophic responses

As previously outlined, WB hypertrophic responses to RE-T were significantly blunted with advancing age (Figure 2D). These hypertrophic responses were not, however, associated with any aspect of baseline physical function (WB, upper body, or lower body strength) or muscle quality (Table 1). In addition, despite younger individuals performing greater absolute work (106 ± 6.9 kg) than both middle-aged (94.3 ± 6.1 kg) and older (86.0 ± 6.1) individuals during RE-T, there was no significant correlation between work and hypertrophy (R^2^ = 0.009; *P* = 0.52).

Baseline characteristics relating to body composition included WB and leg lean mass, RSMI, WB and Ab adiposity, the android/gynoid ratio, and BMD. As with aspects of muscle function, none of these parameters were associated with hypertrophic responses to RE-T (Table 1). Interestingly, there was a significant relationship between WB adipose losses and (WB) hypertrophic responses (R^2^ = 1.36; *P* < 0.01).

Assessed cardiovascular parameters included three indices of BP (SBP, DBP, and MAP), resting heart rate (RHR), leg blood flow (LBF), LVC, and leg vascular resistance (LVR). Contrary to functional and body composition parameters where no relationships with ensuing hypertrophy were observed, all three facets of BP were significantly correlated (negatively) with WB hypertrophy. Similarly, both LVC and LVR were also correlated with hypertrophic responses. Cholesterol/lipid profiles, RHR, and LBF were not associated with hypertrophic responses (Table 1).

Fasting insulin, glucose, and HOMA-IR before RE-T were not correlated with hypertrophic responses to RE-T (Table 1).

There was no correlation between RE-T–induced hypertrophic responses and basal FSR values before or after RE-T (Tables 1 and 2). Similarly, hypertrophic responses were not correlated with FedEx FSR values before or after RE-T (Tables 1 and 2). In addition, hypertrophic responses to RE-T were not associated with FSR responses to FedEx either before (Figure 6C) or after (Figure 6D). In support of this finding, hypertrophic responses were not associated with the phosphorylation of the anabolic proteins P70S6K (Figure 6E) or 4EBP1 (Figure 6F) in response to FedEx, either before or after RE-T.

## Discussion

RE-T remains a cornerstone intervention in relation to exercise for health. Herein, we conducted a sizeable longitudinal, highly controlled RE-T intervention study in three cohorts of younger, middle-aged, and older individuals. Our aim was to determine links between ageing physiology and metabolism in the context of responses to RE-T. In doing so, we highlight the global effects of RE-T as a function of age.

Skeletal muscle mass and function incipiently decline with ageing, albeit in a highly heterogeneous fashion (28). In the present study, we found that, despite all groups being well matched for lean mass (by DXA) at baseline, both middle-aged and older groups failed to demonstrably increase muscle mass — even after 5 months of progressive RE-T. While this age-related failure in muscle hypertrophy has been shown in other studies (8, 18), our current data reveal that this occurs earlier in life than was previously known, i.e., 50–65 years. This suggests that whatever processes drive maladaptation to RE-T with ageing (e.g., endocrine, autocrine/paracrine, mechanotransduction deficiencies, ref. 8), they must also be present in midlife. Despite this lack of muscle hypertrophy, all age groups showed the same relative increase in strength-related performance (1-repetition maximum [1-RM]) in response to RE-T. Indeed, while (cross-sectional) ageing was associated with declines in muscle “mechanical quality” (i.e., thigh mass in relation to 1-RM), RE-T stimulated relatively similar increases in all three age groups. Considering the lack of muscle hypertrophy in middle and older-aged groups — which was not associated with reduced absolute workload, such increases in muscle performance and mechanical quality appear to be driven by factors other than muscle hypertrophy. These likely include neural adaptations relating to learning, coordination, and neural activation — which are reportedly well preserved in ageing (29). Our findings also highlight that age-related declines in muscle function and mechanical quality can occur in the absence of overt muscle atrophy, highlighting the importance of incorporating functional assessments into diagnostics of sarcopenic muscle in ageing (30).

To seek potential explanations for any age-related differences in muscle mass gains with RE-T, we also quantified acute MPS before and after RE-T under conditions of feeding in tandem to a single bout of RE-T. Since the inception of this study, we have shown that MPS responses to acute RE-T (in younger individuals) are unrelated to ensuing muscle hypertrophy following long-term RE-T (31). This fits with our present data, where we found no age-related differences at baseline or correlative relationships between MPS (nor in proxies of mTORc1 signaling) and muscle growth. Interestingly, we did observe that the magnitude of acute MPS in response to feeding plus acute resistance exercise was greater after RE-T in the younger cohort (i.e., those exhibiting hypertrophy) only compared with the middle- and older-aged cohort. As such, it is possible that acute MPS is a more sensitive predictor of RE-T–induced hypertrophy if measured in a period when exercise is more “accustomed.”

Increases in adiposity as a consequence of ageing are associated with a host of risk factors — especially in relation to excess Ab fat (32). As expected, both WB fat and Ab fat increased across age. Interestingly, RE-T reduced WB and Ab body fat in younger and middle-aged but not older adults. As this was not related to work performed (relationship between work and WB adipose losses: R^2^ = 0.002; *P* = 0.77), reductions in body fat with RE-T may be associated with lean mass gains increasing resting energy expenditure (33), rather than the metabolic demands of RE-T per se. On this basis, it is of interest that we identified a negative correlation between RE-T–induced increases in WB lean mass and reductions in WB adiposity. Therefore, despite increasing muscle function and mechanical quality, RE-T was ineffective in reducing body fat in our older cohort, at least in part due to the failure to increase lean mass. Thus, it is likely that alternative training regimens to RE-T (e.g., aerobic exercise, cross-training, summarized in ref. 34) are needed to evoke reductions in body fat in response to exercise in older individuals — especially given the fact that, in our study, higher energy intake could not explain this observation (Table 3).

BMD declines with age, a feature that is associated with increased risk of osteoporosis and osteopenia, and exercise is recommended as a preventive and therapeutic strategy against aging-induced bone weakness. Intriguingly, as for muscle anabolism, the osteogenic potential of ageing bone in response to mechanical loading is also limited (35). Here, we found that cross-sectional ageing was associated with reduced WB BMD but that RE-T did not ameliorate this, nor did RE-T enhance BMD in any age group. While important localized bone remodeling cannot be excluded, it would appear that our 5-month WB regimen was ineffectual in remodeling bone. This could be for several reasons, including the lack of impact of RE-T or duration required for bone remodeling (36). Future studies aimed at modulating BMD (in relation to ageing or otherwise) may consider the use of longer durations or distinct exercise paradigms.

One of the major health benefits of exercise relates to adaptation in the cardiovascular system, e.g., improving endothelial function, lowering BP. While aerobic exercise paradigms are well-established modulators of cardiovascular remodeling and function (37), strength training paradigms can also lower BP (38). In the present study, as expected, MAP increased across age (and in middle- and older-aged groups vs. our younger cohort). However, in response to RE-T, only the middle-aged group exhibited declines in MAP, such that after RE-T these values were indistinguishable from the younger group. The lack of an effect in the younger group is consistent with the notion that they were entirely normotensive at baseline. Although speculative, the lack of modulation of MAP in the older group may relate to the lack of effect of RE-T on adiposity, since adiposity and MAP are strongly linked (39, 40). We also quantified LVC and noted an expected age-related decline (41, 42) but did not find any improvements across the groups. Given that these measures were made in the fasted and rested state, it is likely that RE-T did not overcome increased sympathetic vasoconstrictor activity associated with ageing (42). Nonetheless, this does not mean that endothelial function may not have improved; other techniques, such as flow-mediated dilatation, would be needed to assess this.

We also investigated the effect of age and RE-T upon biomarkers of metabolic health, including fasting glucose/insulin (and thus HOMA-IR), in addition to markers of dyslipidemia, i.e., plasma LDL/HDL cholesterol and triglycerides. HOMA-IR tended to increase with age (due to increases in blood glucose) and was reduced by RE-T, suggesting that where HOMA-IR was elevated in the older group, RE-T is able to mitigate this rise; this is presumably due to the effects of RE-T on glucose uptake and glycogen turnover (43). In this study, both LDL cholesterol and triglycerides also increased as a function of age but were unaffected by RE-T. While it has been suggested that RE-T is a viable alternative to aerobic exercise training to offset dyslipidemia, it is also clear that intense activity is required to elicit reductions in LDL cholesterol and triglyceride levels (44). Indeed, while intense aerobic exercise stimulates the clearance of plasma LDL cholesterol and triglycerides, this is unlikely to be the case with RE-T, maybe explaining the lack of effects in our older group.

As with all studies, we acknowledge some limitations. DXA is not the most accurate measure of skeletal muscle mass in comparison to MRI or creatine stable isotope tracers and has been shown to underestimate hypertrophy (45), meaning that subtle age-related differences at baseline and following RE-T may have been missed. As is common practice for RE-T, the intensity of our progressive RE-T program was based on baseline values of individuals (e.g., %1-RM). Therefore, our older individuals, despite performing the same relative work, performed less absolute work than the young and middle-aged subjects. In addition, the ability to truly assess voluntary strength beyond midlife has been questioned (46), and, although controversial, if correct our 1-RM assessments to determine the RE-T loads may have underestimated the relative workloads in our middle-aged and older groups. As high levels of habitual physical activity may act as a stimulus for muscle remodeling, constant physical activity monitoring would have been advantageous. To try and control for this, all subjects recruited did not participate in regular moderate-high intensity aerobic exercise and none had participated in RE-T for the last 2 years. All subjects were asked to maintain their standard level of physical activity for the duration of the study, with International Physical Activity Questionnaire scores not being significantly different among the age groups at initial screening. Similarly, all subjects were instructed to maintain their normal diet throughout the study to prevent exercise-induced changes, and our diet diaries revealed no major differences in macronutrient or micronutrient intake among the age groups (Table 3). To conclude, we provide a comprehensive data set relating to the effects of RE-T as a function of age, which offers valuable biological insight and that will help pave the way for testing and selecting the most appropriate exercise interventions aimed at improving physiological function and metabolic health in relation to ageing.

## Methods

### Subject characteristics.

We recruited three groups of subjects consisting of young (*n* = 11, 25 ± 4 yr; BMI 24 ± 1 kg/m^2^), middle-aged (*n* = 20, 50 ± 4 yr; BMI 27 ± 1 kg/m^2^), and older (*n* = 17, 70 ± 3 yr; BMI 27 ± 1 kg/m^2^) men and women (~50:50) who were well matched for baseline lean mass (Table 1). All subjects were screened by means of a medical questionnaire, physical examination, and resting ECG, with exclusions for overt muscle wasting (>1 SD below age norms); metabolic, respiratory, or cardiovascular disorders; or other signs and symptoms of ill health. All subjects had normal blood chemistry, were normotensive (BP <140/90), and were not prescribed medication. All subjects performed activities of daily living but did not routinely participate in moderate-to-high intensity aerobic exercise, and none had participated in RE-T in the last 2 years. Subjects were recruited over a 3-year period via poster advertisements in the local community and via age-selected postal invites. All screening and acute study sessions took place at the University of Nottingham Medical School at the Royal Derby Hospital Centre. Intervention delivery (RE-T) occurred at two sites, based on geographical preference of the subjects: Derby College, Derby, United Kingdom, and the University of Nottingham Fitness Centre, Nottingham, United Kingdom. All intervention delivery was supervised by a single member of research staff.

### Acute studies (before and after RE-T).

Subjects were instructed to refrain from exercise for 72 hours prior to each study day and from alcohol and caffeine for 24 hours. Subjects fasted from 2100 hour the night before (water ad libitum) and reported to the laboratory at 0900 hours. Body composition was measured by dual-energy X-ray absorptiometry (DXA; Lunar Prodigy II, GE Medical Systems), with leg muscle mass measured on the dominant leg as the area inferior to the lowest visible point of the coccyx and the Ab area defined as the lowest visible point of the coccyx upward to the highest visible point of the pelvic girdle. All other regions were automatically assigned by the integrated DXA software package (enCORE software, GE Healthcare).

Subjects then had a polyethylene catheter inserted into the antecubital vein of one arm for tracer infusion and the femoral vein of one leg for venous blood sampling. A baseline blood sample was initially taken from the antecubital vein for measures of fasting insulin, glucose, cholesterol, and triglycerides. Plasma insulin concentrations were measured in duplicate using undiluted samples, from blood collected in EDTA-coated collection tubes, on a high-sensitivity human insulin ELISA (DRG Instruments GmbH). Plasma glucose levels were measured in duplicate using undiluted samples, from blood collected in lithium heparin-coated collection tubes, on a blood glucose analyzer (ILAB 300 Plus Clinical Chemistry System). Reports of serum cholesterol and triglyceride profiles were produced by the Clinical Chemistry Department at the Royal Derby Hospital by analysis of undiluted serum samples from blood collected in serum separator tubes. Cholesterol profile results reported total triglycerides, total cholesterol, LDL cholesterol, and HDL cholesterol. Thereafter, blood samples were taken from the femoral vein every 20 minutes throughout the study.

After 70 minutes lying supine, measures of RHR, SBP, and DBP were made using an OMRON (OMRON Healthcare UK Ltd) automated BP monitor (recorded as the mean of 3 measurements). MAP, calculated as two-thirds DBP plus one-third SBP, was also recorded and used to calculate LVC and LVR. LVC (shown as l/min•100 mmHg^–1^) = [LBF (l/min)/MAP (mmHg) × 100]. LVR (shown as l/min•100 mmHg^–1^) = MAP (mmHg)/cardiac output (l/min)/LBF (l/min), where stroke volume was assumed to be 70 ml irrespective of age (47). At this time, basal LBF (femoral artery) measures were made intermittently for 40 minutes. A mean value from 3 measurements was used to obtain the reported basal value, with no significant differences between the 3 measurements made in any subject. LBF was measured using Doppler ultrasound (Toshiba Nemio-17, Toshiba Medical Systems), with a single 5-MHz frequency probe to measure mean blood velocity (MBV) and arterial lumen diameter in the common femoral artery. Measurements were made 2 to 3 cm proximal to the bifurcation of the femoral artery to minimize the effect of turbulence; the insonation angle was <60°. Arterial lumen diameter (mm) was measured by video calipers for each measurement and was defined as the maximum distance between the media-adventitia interface of the near wall and the lumen-intima interface of the far wall of the vessel. LBF (l/min) was calculated as follows: (MBV [cm/s] × π × [femoral artery radius {mm^2^}])/1,000 × 60.

A primed, continuous infusion (0.66 mg/kg, 1 mg/kg/h) of [1, 2-^13^C_2_] leucine (99 atoms percentage excess; Cambridge Isotopes Ltd.) was started at 0 hours and maintained for the duration of the study, with an increase (to 1.2 mg/kg/h) when nutrition was first provided (at 130 minutes) to prevent dilution of tracer. Muscle biopsies of *m*. *vastus lateralis* were taken under sterile conditions at 0, 120, and 250 minutes using the conchotome biopsy technique (48), with 1% lidocaine (B. Braun Melsungen) as local anesthetic. Muscle was rapidly dissected free of fat and connective tissue, washed in ice-cold saline, and snap frozen in liquid N_2_ before storage at –80°C until further analysis.

After the second biopsy at 120 minutes, subjects performed 6 × 8 repetitions of full-cycle leg extensions at 75% 1-RM on a free-standing machine (ISO Leg Extension, Leisure Lines GB Ltd.). Immediately after exercise (at ~130 min), the subjects received, over 2 hours, an oral feed (Fortisip, Nutricia Clinical Care), which supplied energy at 4.25 times basal metabolic rate, as calculated by standard equations (17). The feed had a composition similar to that of a normal mixed meal (16% protein, 49% carbohydrate, and 35% fat) and was given as a priming bolus (3 doses immediately after exercise), with 4 further doses every 30 minutes thereafter. Doses were between 61 and 96 ml, based upon subject body weight to provide 6.5 kJ/kg body weight/30 minutes. This protocol was repeated at least 3, but less than 7, days after the subjects last training bout at the end of the supervised 20-week WB RE-T regime (Figure 1). This allowed us to study the effects of RE-T rather than any lingering remaining acute effects of a single exercise RE-T session (49).

During the tenth week of RE-T subjects were asked to maintain a 4-day diet diary (adapted from the Royal Derby Hospital dietetic department) of all food and drink consumed, with 2 of the 4 days to be days on which a RE-T session occurred. Diet diaries were analyzed using Microdiet version 5 (Downlee Systems Ltd.).

### RE-T.

The fully supervised RE-T program was designed to achieve skeletal muscle hypertrophy and improvements in strength, based on previously published recommendations for exercise intensity and duration (50). Subjects trained 3 times per week, with each session lasting approximately 60 minutes. During 4 weeks of induction training (to ensure adoption of and adherence to correct technique) intensity was increased from 40% to 60% 1-RM. For the remaining 16 weeks of training, intensity was set at 70% 1-RM, with multiple sets of 12 repetitions and 2 minutes of rest between sets. The same number of repetitions were performed each session over a total of 8 exercises; seated chest press, latissimus pull down, seated lever row, leg extension, leg curl, leg press, back extension, and Ab curl. 1-RM assessments were made every 4 weeks to ensure the intensity of training remained constant, i.e., 70% 1-RM, to account for strength improvements. Subjects were to be excluded from the study for noncompliance, which was defined as follows: nonattendance for >6 consecutive sessions, less than 75% attendance, or failure to complete the set exercise regime on >15% attendance; although no subject was excluded. WB strength was determined as the sum of force produced by three lower body exercises, leg extension, leg curl, and leg press, and three upper body exercises, latissimus pull down, lever seated row, and seated chest press, with Newtons calculated as weight lifted × 9.807 based on a standard gravitational field. Upper and lower body strength was determined by the sum of force produced by the three exercises relative to that body region. Muscle quality was determined as lower body strength per gram lean leg mass.

### MPS.

Approximately 20 of muscle tissue from each biopsy was used for measures of MPS, as previously described (51). In brief, the myofibrillar pellet was precipitated by homogenization and centrifugation with the soluble myofibrillar protein and then precipitated by perchloric acid. Protein-bound aa were released by acid hydrolysis in Dowex H^+^ resin (Sigma-Aldrich) slurry before being purified by ion exchange chromatography on Dowex H^+^ resin. The aa were then derivatized as their N-acetyl-N-propyl esters as previously described (52). Incorporation of [1,2-^13^C_2_] leucine into protein was determined by gas chromatography–combustion-isotope ratio mass spectrometry (Delta Plus XP, Thermofisher Scientific) using our standard techniques (53), with the FSR of myofibrillar protein determined from the incorporation of [1, 2-^13^C_2_], using the precursor labeling of venous α-KIC between subsequent muscle biopsies (54). Plasma phenylalanine concentrations were measured via our standard techniques using a ^2^H_2_ phenylalanine internal standard, with reference to a standard curve of known concentration (55).

### Immunoblotting.

To investigate the possible effects of age and/or RE-T on anabolic signaling, we measured protein phosphorylation of mechanistic target of rapamycin (mTORc1) substrates 4EBP1 and P70S6K1 (as likely indicators of such activity) in response to acute exercise plus feeding. The supernatant (sarcoplasmic fraction) obtained from the myofibrillar preparation described above was standardized to a protein concentration of 1 mg/ml by dilution with Laemmli buffer, mixed, and heated at 95°C for 5 minutes, before 15 μg of protein/lane was loaded on to Criterion XT Bis-Tris 12% SDS-PAGE gels (Bio-Rad) for electrophoresis at 200 V for 60 minutes. Proteins were electroblotted on to 0.2 μm PVDF membranes (Bio-Rad) at 100 V for 30 minutes, and membranes were blocked in 5% low-fat milk in TBS-T (Tris-buffered saline and 0.1% Tween-20; both Sigma-Aldrich) for 1 hour; membranes were rotated overnight with primary antibody (Cell Signaling Technology) at 1:2,000 at 4°C. Membranes were washed (3 × 5 min) with TBS-T and incubated for 60 minutes at room temperature with HRP-conjugated anti-rabbit secondary antibody (Cell Signaling Technology), before further washing (3 × 5 min) with TBS-T and incubation for 5 minutes with ECL reagents (Enhanced chemiluminescence kit; Immunstar; Bio-Rad). Blots were imaged and quantified by peak density within the linear range using the Chemidoc XRS system (Bio-Rad). Coomassie staining was used to correct for loading (56).

### Statistics.

Statistical analyses were performed using Graph Pad Prism version 6.00. All data are reported as mean ± SEM, with significance set at *P* < 0.05. Two-tailed Student’s *t* tests and 2-way rANOVA with Tukey post-hoc analysis were used to compare before and after training values and differences among the age groups, respectively. One-way ANOVA with Tukey post-hoc analysis was used to compare of responses to RE-T among the age groups. Pearson’s correlation was used to explore relationships between physiological parameters (i.e., adiposity) and/or responses to RE-T (i.e., hypertrophy).

### Study approval.

This study was reviewed and approved by the University of Nottingham Medical School Ethics Committee (D/2/2006) and complied with the Declaration of Helsinki. All subjects gave written informed consent to participate in the study prior to inclusion after all procedures and risks were explained.

## Author contributions

PLG, KS, and PJA were responsible for designing the studies. BEP and JPW were responsible for conducting the studies. BEP and KS were responsible for acquiring the data. BEP, KS, and PJA were responsible for analyzing the data. BEP was responsible for drafting the manuscript. All authors contributed to and approved the final manuscript version.

## Supplementary Material

ICMJE disclosure forms

## Figures and Tables

**Table 3:**
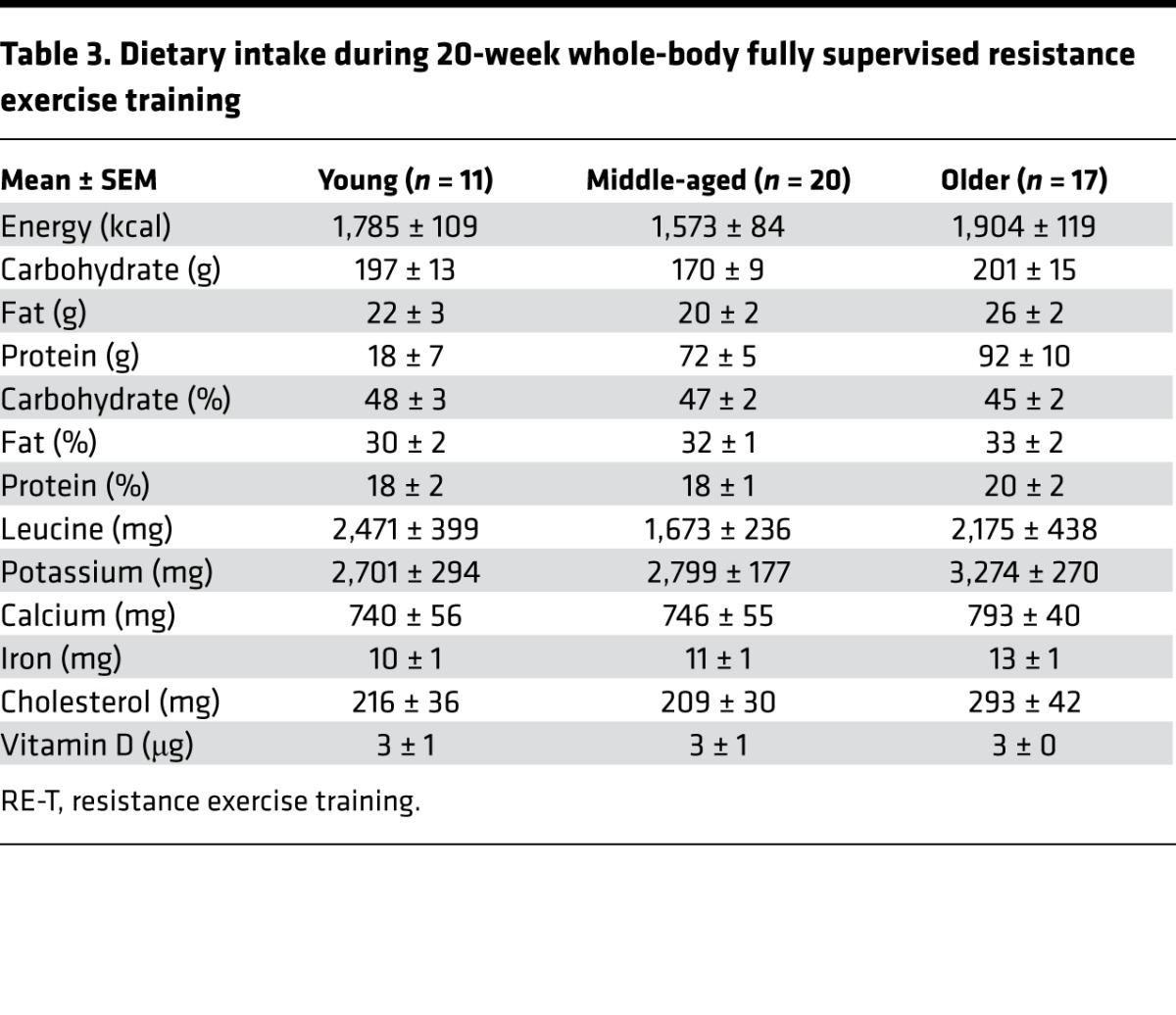
Dietary intake during 20-week whole-body fully supervised resistance exercise training

**Table 2:**
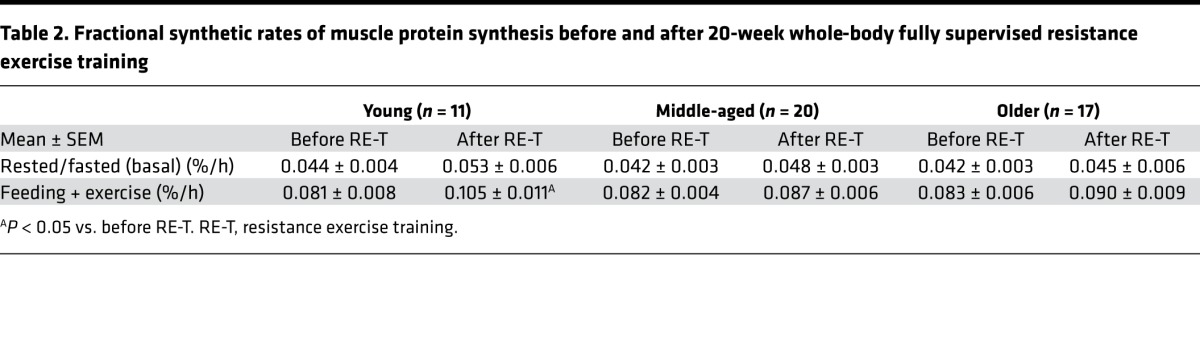
Fractional synthetic rates of muscle protein synthesis before and after 20-week whole-body fully supervised resistance exercise training

**Table 1:**
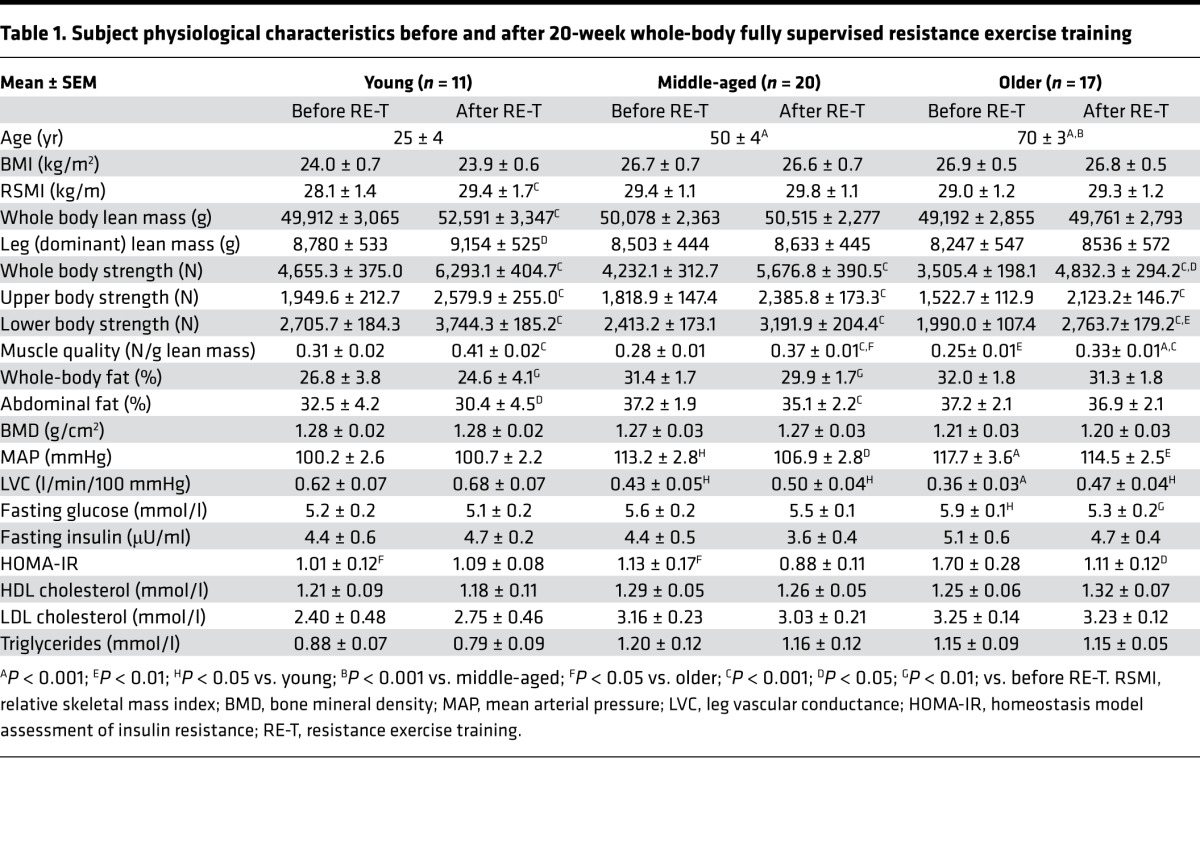
Subject physiological characteristics before and after 20-week whole-body fully supervised resistance exercise training

## Version 1 08/31/2017

Electronic publication
